# Mosquito-Disseminated Pyriproxyfen Yields High Breeding-Site Coverage and Boosts Juvenile Mosquito Mortality at the Neighborhood Scale

**DOI:** 10.1371/journal.pntd.0003702

**Published:** 2015-04-07

**Authors:** Fernando Abad-Franch, Elvira Zamora-Perea, Gonçalo Ferraz, Samael D. Padilla-Torres, Sérgio L. B. Luz

**Affiliations:** 1 Laboratório de Ecologia de Doenças Transmissíveis na Amazônia, Instituto Leônidas e Maria Deane—Fiocruz Amazônia, Manaus, Brazil; 2 Departamento de Ecologia, Instituto de Biociências, Universidade Federal do Rio Grande do Sul, Porto Alegre, Brazil; 3 Biological Dynamics of Forest Fragments Project, Smithsonian Tropical Research Institute/Instituto Nacional de Pesquisas da Amazônia, Manaus, Brazil; Centers for Disease Control and Prevention, UNITED STATES

## Abstract

**Background:**

Mosquito-borne pathogens pose major public health challenges worldwide. With vaccines or effective drugs still unavailable for most such pathogens, disease prevention heavily relies on vector control. To date, however, mosquito control has proven difficult, with low breeding-site coverage during control campaigns identified as a major drawback. A novel tactic exploits the egg-laying behavior of mosquitoes to have them disseminate tiny particles of a potent larvicide, pyriproxyfen (PPF), from resting to breeding sites, thus improving coverage. This approach has yielded promising results at small spatial scales, but its wider applicability remains unclear.

**Methodology/Principal Findings:**

We conducted a four-month trial within a 20-month study to investigate mosquito-driven dissemination of PPF dust-particles from 100 ‘dissemination stations’ (DSs) deployed in a 7-ha sub-area to surveillance dwellings and sentinel breeding sites (SBSs) distributed over an urban neighborhood of about 50 ha. We assessed the impact of the trial by measuring juvenile mosquito mortality and adult mosquito emergence in each SBS-month. Using data from 1,075 dwelling-months, 2,988 SBS-months, and 29,922 individual mosquitoes, we show that mosquito-disseminated PPF yielded high coverage of dwellings (up to 100%) and SBSs (up to 94.3%). Juvenile mosquito mortality in SBSs (about 4% at baseline) increased by over one order of magnitude during PPF dissemination (about 75%). This led to a >10-fold decrease of adult mosquito emergence from SBSs, from approximately 1,000–3,000 adults/month before to about 100 adults/month during PPF dissemination.

**Conclusions/Significance:**

By expanding breeding-site coverage and boosting juvenile mosquito mortality, a strategy based on mosquito-disseminated PPF has potential to substantially enhance mosquito control. Sharp declines in adult mosquito emergence can lower vector/host ratios, reducing the risk of disease outbreaks. This approach is a very promising complement to current and novel mosquito control strategies; it will probably be especially relevant for the control of urban disease vectors, such as *Aedes* and *Culex* species, that often cause large epidemics.

## Author Summary

Mosquito-transmitted diseases are among the most challenging infectious threats worldwide. Mosquito control is crucial for preventing infection and disease, particularly when effective vaccines or drugs are unavailable. A major drawback of current mosquito control strategies is that mosquito breeding sites are often overlooked, and therefore left untreated, during control campaigns. One appealing alternative proposes exploiting the innate breeding-site–finding ability of female mosquitoes to have them disseminate tiny insecticide particles that poison their offspring. Thus far, however, this idea has only been tested in small-scale trials. Here we show that mosquitoes effectively transferred insecticide particles from dissemination stations to sentinel breeding sites over distances between 3 and 400 m in a tropical urban neighborhood. This yielded high breeding-site coverage, with up to 94.3% of sentinel breeding sites presenting evidence of contamination with mosquito-disseminated insecticide. We recorded a 10-fold increase of juvenile mosquito mortality and a 10-fold decrease of adult mosquito emergence during the four-month dissemination trial. In combination with other tactics, this approach has the potential to considerably enhance mosquito-borne disease prevention, particularly in urban settings.

## Introduction

Mosquito-borne infectious diseases pose major public health challenges worldwide. Malaria and dengue are the most widespread, but other pathogens are also of concern, including viruses such as West Nile, chikungunya or Japanese encephalitis, and parasites such as those causing filariasis [1–5]. Urban vectors are especially problematic because they can transmit pathogens to large populations of susceptible humans, causing epidemics [1,2]. Since effective vaccines or treatments are available for only a few mosquito-borne diseases, prevention heavily relies on vector control; to date, however, mosquito control has proven difficult [1–3,6–9]. In particular with *Aedes aegypti* and *Ae*. *albopictus* (the vectors of dengue, chikungunya, and yellow fever), current strategies depend on the ability of mosquito control staff to detect and eliminate mosquito breeding sites in and around human residences [3,8]. Unfortunately, both *Aedes* species breed in small water-holding containers that can be difficult to detect, leading to low breeding-site coverage in control campaigns; this partially explains why the performance of such campaigns can be so poor [9]. In general, mosquito control tactics that rely on source reduction via larval habitat management all face the challenge of low coverage, whereby cryptic or inaccessible mosquito breeding sites remain untreated [3,8].

Proof-of-concept research has shown that the egg-laying behavior of female mosquitoes can be exploited to have them disseminate tiny particles of pyriproxyfen (PPF), a potent larvicide, from resting sites to nearby breeding sites [10,11]. This strategy relies on the innate ability of female mosquitoes to find and reach suitable breeding sites and on the ‘skip-oviposition’ behavior of some species, whose females visit several breeding sites to lay a few eggs in each [12,13]. The idea entails luring mosquitoes to ‘dissemination stations’ treated with PPF dust-particles that adhere to the insect’s body and are thus transferred to clean breeding sites subsequently visited for oviposition [10].

Although appealing, this approach has only been tested in small areas, with PPF dissemination measured at very short distances [10,11,14–16]; recently, a larger trial (ref. [17]) used an emulsifiable-PPF spray instead of dust-particle dissemination stations. Here, we investigate whether adult mosquitoes can transfer PPF particles from lure dissemination stations to sentinel breeding sites in a tropical neighborhood, and assess the impact of mosquito-disseminated PPF on juvenile mosquito mortality and adult mosquito emergence.

## Methods

### Ethics statement

All field procedures were carried out with permission from dwelling owners. Sérgio LB Luz holds a permanent license (27733–1) from the Brazilian Institute for the Environment and Natural Resources (IBAMA) for sampling disease vectors.

### Study site

**Fig 1:**
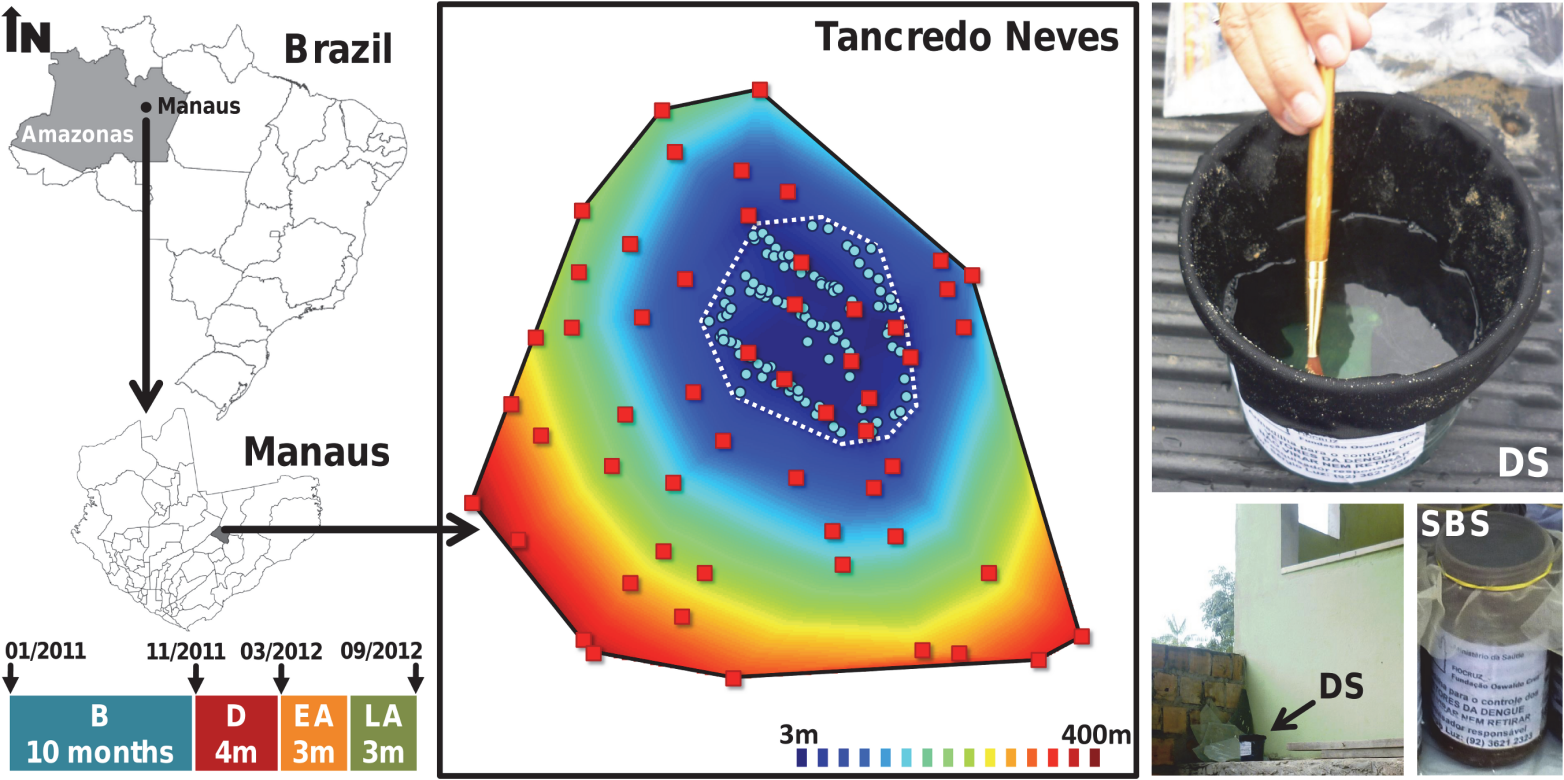
Study area in the *Tancredo Neves* neighborhood, Manaus, state of Amazonas, Brazil. One hundred pyriproxyfen dissemination stations (DSs; pale blue circles) were deployed over four months in an intervention sub-area (dotted polygon). Sentinel breeding sites (SBSs) in surveillance dwellings (red squares) were 3 to 397 m from the nearest DS (100 blue-red distance contours are shown). Colored bars represent study periods: B, before; D, during; EA, early after; and LA, late after the trial; period duration in months and key dates along the study timeline are also given.

The study took place at the *Tancredo Neves* neighborhood in the city of Manaus, Amazonas, Brazil (3°6’S, 60°1’W; Fig 1). *Tancredo Neves* is a lower middle-class residential neighborhood where most people live in single-family houses with a small yard; house/yard compounds will be referred to as ‘dwellings’ hereafter. *Aedes aegypti* and *Ae*. *albopictus* infest most dwellings in the study area, where dengue cases are common [9].

### Mosquito surveillance

We set up a mosquito-surveillance network spanning about 50 ha of *Tancredo Neves* and comprising 55 randomly selected dwellings. The presence of egg-laying mosquitoes in each dwelling was sampled via ‘sentinel breeding sites’ (SBSs). SBSs were 580-ml dark-brown plastic cups (Fig 1) baited with 200 ml of hay infusion (approximately 5g dry *Zoysia* sp./liter of tap water, fermented for six days in a closed plastic container) diluted in 250ml of tap water. As for standard ovitraps, SBSs offer artificial breeding habitats that can be promptly set/checked and from which larvae can be removed for further analysis; we used screw-capped cups to avoid any spillover of SBS contents (hence possible cross-contamination) during SBS retrieval and transportation to the laboratory. Mosquito surveillance was run monthly from January 2011 to September 2012 (see study timeline in Fig 1) by simultaneously setting three SBSs in each dwelling during six days per month. We coded each SBS individually to ensure that each was set always at the same location; SBSs were thoroughly washed with water and soap between monthly sampling rounds. Any missing SBS was replaced by a new one with a different code in the next sampling round. Overall, we analyzed dwelling-level data from 1,075 dwelling-months (excluding dwellings that were unavailable for sampling at certain months) and breeding site-level data from 2,988 SBS-months (excluding SBSs that did not produce data in a given month because they were overturned, went missing, or corresponded to dwellings that were unavailable for sampling). Dwelling-level data allowed us to investigate whether mosquitoes would effectively disseminate PPF over the whole study area, while SBS-level data provided insight on (i) breeding-site coverage (as measured by the fraction of SBSs that became contaminated with mosquito-disseminated PPF) and (ii) the effects of the trial on juvenile mosquito mortality and adult mosquito emergence (see below).

### Mosquito mortality and emergence

SBSs were retrieved after six days of operation to avoid emergence of adult mosquitoes in the study dwellings. Once in the laboratory, the contents of each SBS were transferred to a white plastic cup to ease the observation of mosquito juveniles; each cup received the same code as the corresponding SBS and was capped with gauze and kept for 8–16 days to monitor mosquito development. A pinch of TetraMin fish food (Tetra, Melle, Germany) was added every other day to each cup. Mosquito larvae found in each individual SBS-month were identified as *Ae*. *aegypti*, *Ae*. *albopictus* or *Culex* spp. [18] (we ignore a few, rarer taxa in the present analyses), and were checked every two days to score juvenile mosquito death or adult mosquito emergence. For each SBS-month, juvenile mortality was estimated as the percent of individuals that died as larvae or pupae, and adult mosquito emergence as the sum of all individuals that emerged as adults.

### Intervention

‘Dissemination stations’ (DSs; Fig 1) were two-liter, black plastic cups with 400 ml of tap water and the inner wall lined with black, velvet-like cloth dusted with 5 g/m^2^ of PPF (Sumilarv 0.5 g granules, Sumitomo, London, UK) ground to fine powder in a metal mortar. PPF is an insect juvenile-hormone analog that kills immature mosquitoes, especially pupae, at extremely low doses; it also reduces fertility in adult mosquitoes, but has no lethal or repellent effects on them [10,16]. PPF is recommended by the World Health Organization as a safe mosquito control means even in drinking water [3], and is currently endorsed by the Brazilian Ministry of Health [8]. One hundred DSs were deployed in a sub-area of about 7 ha nested within the study area; DSs were 3 to 397 m from the nearest SBS (Fig 1). DS deployment (‘the trial’ hereafter) took place from December 2011 (month 11) to March 2012 (month 14), coinciding with the rainy season [9]; all DSs were removed from the field at the end of month 14 (Fig 1). DSs were placed in sheltered locations and checked fortnightly throughout the four months of the trial to refill water, re-dust cloth with PPF, and replace lost cups.

In the laboratory, individual SBSs were scored each month by one of the investigators (Elvira Zamora-Perea) as contaminated or not contaminated with PPF. Contamination was inferred when mosquito juveniles in the SBS developed the abnormal morphology and coloration that characterizes PPF poisoning (large bodies, blackish color; see S1 Fig). In addition to inducing these marked morphological abnormalities, PPF increases larval development time from the typical 8–9 days until adult emergence to 14–16 days until (usually) pupal death. We pre-tested the effectiveness of our PPF in a double-blind, randomized, controlled laboratory trial using 30 independent cohorts of 20 *Ae*. *aegypti* larvae each. All juveniles in the 15 cohorts treated with PPF (0.05 ppm a.i.) died over three weeks of monitoring, whereas only one larva died in the 15 control cohorts (see S1 Text). Juveniles in treated cohorts developed the morphological abnormalities typical of PPF poisoning (see S1 Fig).

### Data analyses

After exploratory/descriptive analyses, we used generalized linear models (GLMs; binomial family, logit link) to analyze binary outcome data (i) at the dwelling level and (ii) at the breeding-site level. At the dwelling level, the binary outcome was 1 for dwellings with at least one SBS presenting evidence of contamination with mosquito-disseminated PPF and 0 otherwise. At the breeding-site level, the binary outcome was 1 for SBSs with evidence of contamination and 0 for those without. We investigated the effects of two key predictors: (a) time-period, comparing 10 months ‘before’, 4 months ‘during’, 3 months ‘early after’, and 3 months ‘late after’ the trial; and (b) log_10_-distance (in meters) between each dwelling and the nearest DS, which was also used to approximate the distance between SBSs set in each dwelling and the nearest DS (see Fig 1 for timeline and spatial arrangement of DSs and dwellings). Because no PPF was present in the environment before the trial, we used Firth’s correction [19] to estimate period effects with ‘before the trial’ as reference level. ‘Distance*period’ interactions were also tested. Dwelling-level models adjusted for the number of SBSs that were operational in each dwelling and month, which was specified as a three-level categorical covariate (1, 2, or 3 operational SBSs, with ‘1 SBS’ as the reference level). Relative model performance was assessed using second-order Akaike information criterion (AICc) scores and related metrics (ref. [20] and S1 Text); likelihood-ratio tests were used to evaluate covariate contribution to model fit. Categorical variables were analyzed with Pearson χ^2^ tests or conditional maximum-likelihood odds ratios [21]. Crude juvenile mortality rates were compared with nonparametric Kruskal-Wallis rank-sum and *post hoc* Tukey tests. Contour plots were built to spatially visualize GLM predictions and juvenile mosquito mortality data. We used linear regression to illustrate the effect of distance to the nearest DS on juvenile mosquito mortality. We analyzed the data using JMP 9.0 (SAS Institute, Cary, NC).

## Results

### Dwelling-level PPF dissemination

All surveillance dwellings presented evidence of contamination with mosquito-disseminated PPF in ≥1 SBS at some time-point during DS deployment. There was evidence of contamination in 75.5%, 80%, 100%, and 94.4% of surveillance dwellings in months 11, 12, 13, and 14, respectively. Afterwards, dwelling-level PPF coverage fell from 79.2–81.5% (months 15–16) to 1.9% (month 20) of dwellings. Table 1 summarizes dwelling-level data over the four study periods.

**Table 1:** Evidence of sentinel breeding site (SBS) contamination with mosquito-disseminated pyriproxyfen: results at the dwelling level.

Trial period	Evidence of contamination		%	95%CI	
	Yes*	No			
Before (months 1–10)	0	540	0.00	0.00	0.55
During (months 11–14)	188	27	87.44	82.50	91.39
Early after (months 15–17)	115	45	71.88	64.53	78.43
Late after (months 18–20)	21	139	13.13	8.53	19.04

*Dwelling-months with at least one contaminated SBS

Pearson χ^2^ = 722.6, d.f. = 3, *P*<0.0001

**Fig 2:**
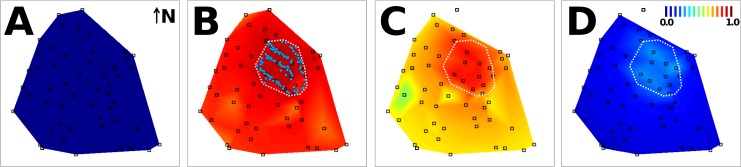
Mosquitoes effectively disseminated insecticide particles at the neighborhood scale. The contour plots are a spatial representation of the (period-specific) probability that at least one sentinel breeding site in a surveillance dwelling had evidence of contamination with mosquito-disseminated pyriproxyfen, as predicted by the generalized linear model in Table 2: A, before; B, during; C, early after; and D, late after the trial. The intervention sub-area is outlined in white in panels B (with pyriproxyfen dissemination stations represented by blue dots) to D. The scale in panel D shows the color code of probabilities. Surveillance dwellings are represented by squares; note that surveillance was discontinued in the northernmost dwelling.

GLMs revealed strong period effects and a negative effect of distance; ‘distance*period’ interactions were not significant. According to the main-effects GLM (Table 2), the odds that a dwelling had evidence of contamination were 96.9 times higher during than before the trial (likelihood-ratio test, χ^2^ = 692.8, d.f. = 1, *P*<0.0001; Table 2, Fig 2). A substantial decline in contamination odds was detected only 4–6 months after DSs were removed (Table 2, Fig 2). The odds of contamination decreased at an average rate of 54.5% for each 10-fold increase in distance between dwellings and DSs (Table 2). A model with only period effects provided a poor fit to the data (ΔAICc = 11.87), but a distance-only model performed much worse and similarly to the intercept-only model (see S1 Text); thus, while both covariates substantially helped explain the data, period effects were more important than distance effects.

**Table 2:** Dwellings with at least one sentinel breeding site-month contaminated with mosquito-disseminated pyriproxyfen: distance and period effects as estimated with a generalized linear model adjusting for the number of sentinel breeding sites that were operational in each dwelling and month (*n* = 1,075 dwelling-months).

Term	Estimate	95%CI	
Intercept	4.992	3.581	7.532
Distance*	−0.787	−1.235	−0.361
Trial period			
Before (months 1–10)	(Reference)		
During (months 11–14)	4.574	3.580	6.998
Early after (months 15–17)	4.020	3.038	6.442
Late after (months 18–20)	2.546	1.549	4.970
Number of operational SBSs			
1	(Reference)		
2	0.346	−0.219	0.932
3	0.699	0.244	1.156

*Log_10_-transformed distance from the nearest dissemination station, in meters

SBS, sentinel breeding site

### Breeding site-level PPF dissemination

**Fig 3:**
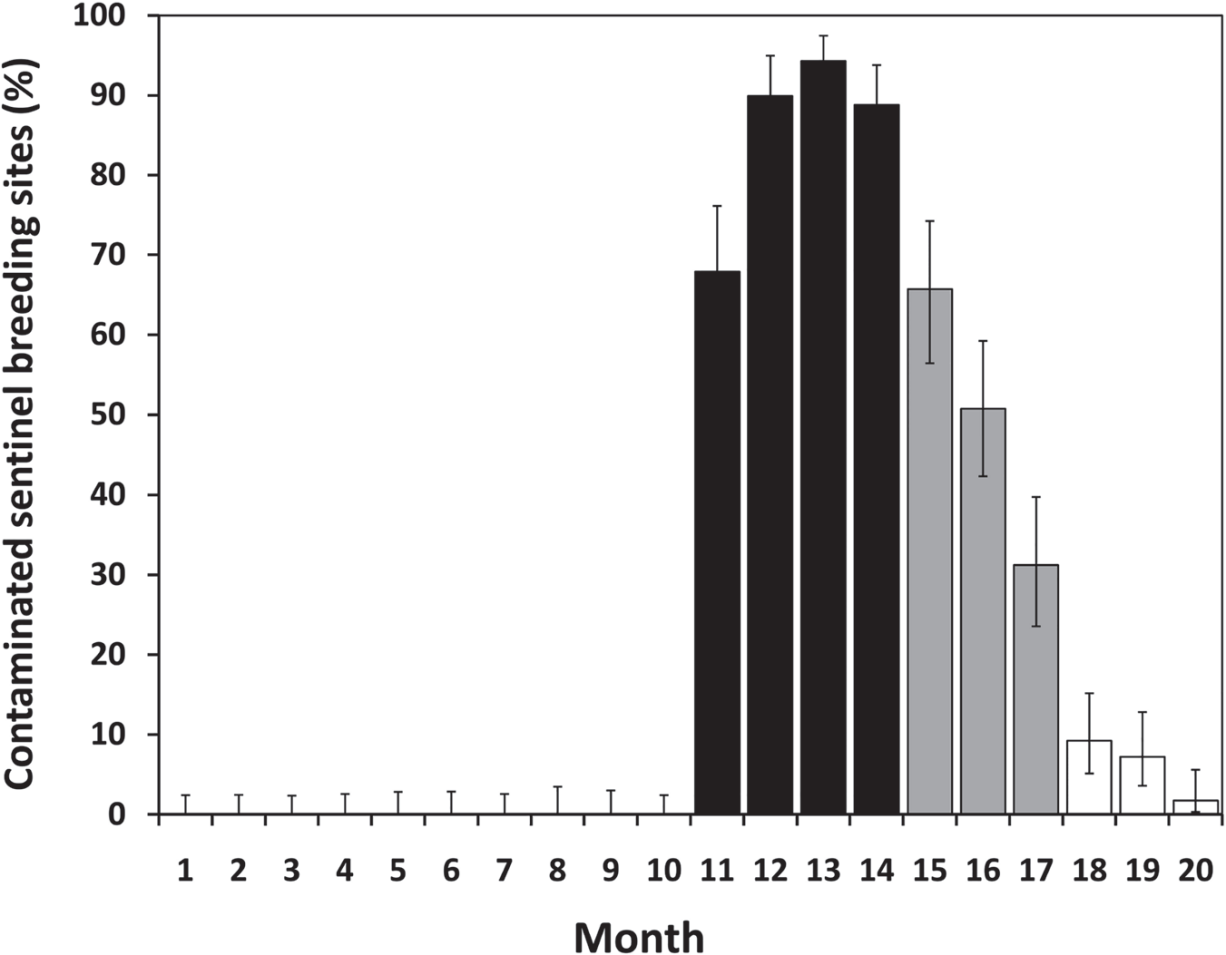
Egg-laying mosquitoes consistently contaminated most sentinel breeding sites (SBSs). Percentage of SBSs that contained ≥1 mosquito larva (*n* = 2,287 SBS-months) and showed evidence of contamination with mosquito-disseminated pyriproxyfen over four periods: before (months 1–10), during (black bars), early after (gray bars), and late after the trial (white bars). Error bars are 95%Cis.

Most of the SBSs that contained larvae (i.e., were visited by ≥1 egg-laying mosquito) consistently became contaminated during the trial: from 67.9% in month 11 to 94.3% in month 13. Afterwards, contamination fell back to 65.7% in month 15 and 1.7% in month 20 (Fig 3). Overall, we found evidence of PPF contamination in >85% of SBSs that were visited by egg-laying mosquitoes during the four-month trial period, with a steep decline afterwards (Table 3).

**Table 3:** Evidence of contamination with mosquito-disseminated pyriproxyfen at the sentinel breeding site (SBS) level.

Trial period	Evidence of contamination*		%	95%CI	
	Yes	No			
Before (months 1–10)	0	1124	0.00	0.00	0.26
During (months 11–14)	364	63	85.25	81.64	88.37
Early after (months 15–17)	177	188	48.49	43.39	53.62
Late after (months 18–20)	23	348	6.20	4.07	9.01

*In 2,287 SBS-months with ≥1 larva

Pearson χ^2^ = 1,391.1, d.f. = 3, *P*<0.0001

**Fig 4:**
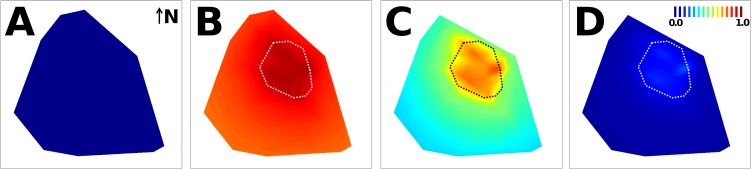
Mosquitoes effectively transferred insecticide particles from dissemination stations to sentinel breeding sites (SBSs) at the neighborhood scale. The contour plots are a spatial representation of the (period-specific) probability that SBSs in surveillance dwellings had evidence of contamination with mosquito-disseminated pyriproxyfen, as predicted by a generalized linear model (Table 4): A, before; B, during; C, early after; and D, late after the trial. The intervention sub-area is highlighted by a dotted polygon; the probability scale bar is presented in panel D. Surveillance dwellings (see Figs 1 and 2) were omitted for clarity.

GLMs revealed period and distance effects similar to those seen at the dwelling level (see Tables 2 and 4). ‘Distance*period’ interactions were, again, not significant; the main-effects model is presented in Table 4, and its spatially-plotted predictions are shown in Fig 4. AICc clearly favored this model over simpler versions (S1 Text).

**Table 4:** Sentinel breeding sites (SBSs) with evidence of contamination with mosquito-disseminated pyriproxyfen: distance and period effects as estimated with a generalized linear model (*n* = 2,287 SBS-months with ≥1 larva).

Term	Estimate	95%CI	
Intercept	5.502	4.267	7.983
Distance*	−1.065	−1.384	−0.759
Trial period			
Before (months 1–10)	(Reference)		
During (months 11–14)	4.886	3.911	7.306
Early after (months 15–17)	3.942	2.975	6.361
Late after (months 18–20)	2.490	1.500	4.912

*Log_10_-transformed distance from the nearest dissemination station, in meters

### Species-specific PPF dissemination

Due to the abundance and oviposition behavior of *Ae*. *aegypti*, we hypothesized that, during the trial, SBSs with larvae of this species would have higher odds of presenting evidence of PPF contamination than SBSs without. Such odds were 329% higher in SBSs with *Ae*. *aegypti* larvae (91.2% contaminated) than in those with only *Ae*. *albopictus* and/or *Culex* spp. larvae (70.5% contaminated; odds ratio 4.29, 95%CI 2.47–7.54). A similar effect was recorded when comparing SBSs with *Ae*. *aegypti* larvae only (i.e., probably visited only by *Ae*. *aegypti*) *vs*. those without (odds ratio 3.42, 95%CI 1.89–6.41); see details in S1 Table.

### Juvenile mosquito mortality

Juvenile mortality was assessed based on 29,922 mosquito larvae/pupae present in 2,287 SBS-months; overall, 9.2% of those mosquitoes (95%CI 8.9–9.5%) died as juveniles. Before PPF dissemination, overall larval/pupal mortalities in SBSs were approximately 2.0/0.1% (*Ae*. *aegypti*), 1.5/0.2% (*Ae*. *albopictus*), and 6.9/0% (*Culex* spp.). During the trial, these figures reached peak values of 27.9/80.7%, 43.6/70%, and 16.7/54.8%, respectively; pre-trial values were restored by months 15–16 (see S1 Dataset).

Before the trial, species-pooled mean juvenile mortality across SBSs was 4.2% (SE = 0.5); 0% mortality was recorded in 87.8% of SBSs with ≥1 larva (data from 1,124 SBSs and 11,970 mosquitoes). Mean juvenile mortality across SBSs rose to 75.1% (SE = 1.8) during the four months of PPF dissemination, with 100% mortality recorded in 61.6% of SBSs with ≥1 larva (data from 427 SBSs and 2,392 mosquitoes). Mean juvenile mortality progressively declined afterwards to 15.8% early after (*n* = 365 SBSs) and to just 0.6% late after the trial (*n* = 371 SBSs).

Mean monthly mortality of *Ae*. *aegypti* juveniles in SBSs rose from a 0–10% range before the trial (median and inter-quartile range [IQR] all 0%) to 62–94% during the trial (median and IQR all 100%), and fell back to 0.3–1.4% (median and IQR all 0%) in the final 3-month period. *Ae*. *albopictus* mean monthly juvenile mortality was <2% (range across months 0–4.6%) before and about 64% (range 29.5–84.2%) during dissemination, and quickly fell back to baseline values after the end of the trial. Juvenile mosquito mortalities were significantly different across the four study periods: Kruskal-Wallis test of species-pooled mortality, χ^2^ = 1,140.5 (d.f. = 3, *P*<0.0001). Tukey tests suggested, however, that *Ae*. *aegypti* mortality was comparable before and late after the trial, with a marginally significant difference when considering all species (see details in S2 and S3 Tables).

Juvenile mosquito mortality was over 20 times higher in SBSs with evidence of PPF contamination (mean across months 67.3%; SE = 1.6) than in SBSs without such evidence (2.8%; SE = 0.3); 100% mortality was recorded in 287 of 564 contaminated SBSs (50.9%; 95%CI 46.8–55%) and in 17 of 1,723 non-contaminated SBSs (1%; 95%CI 0.6–1.5%). During the four months of PPF dissemination, mean mortality in SBSs with evidence of PPF contamination reached 87.9% (*n* = 364 SBSs), *vs*. 0.8% in SBSs without such evidence (*n* = 63).

**Fig 5:**
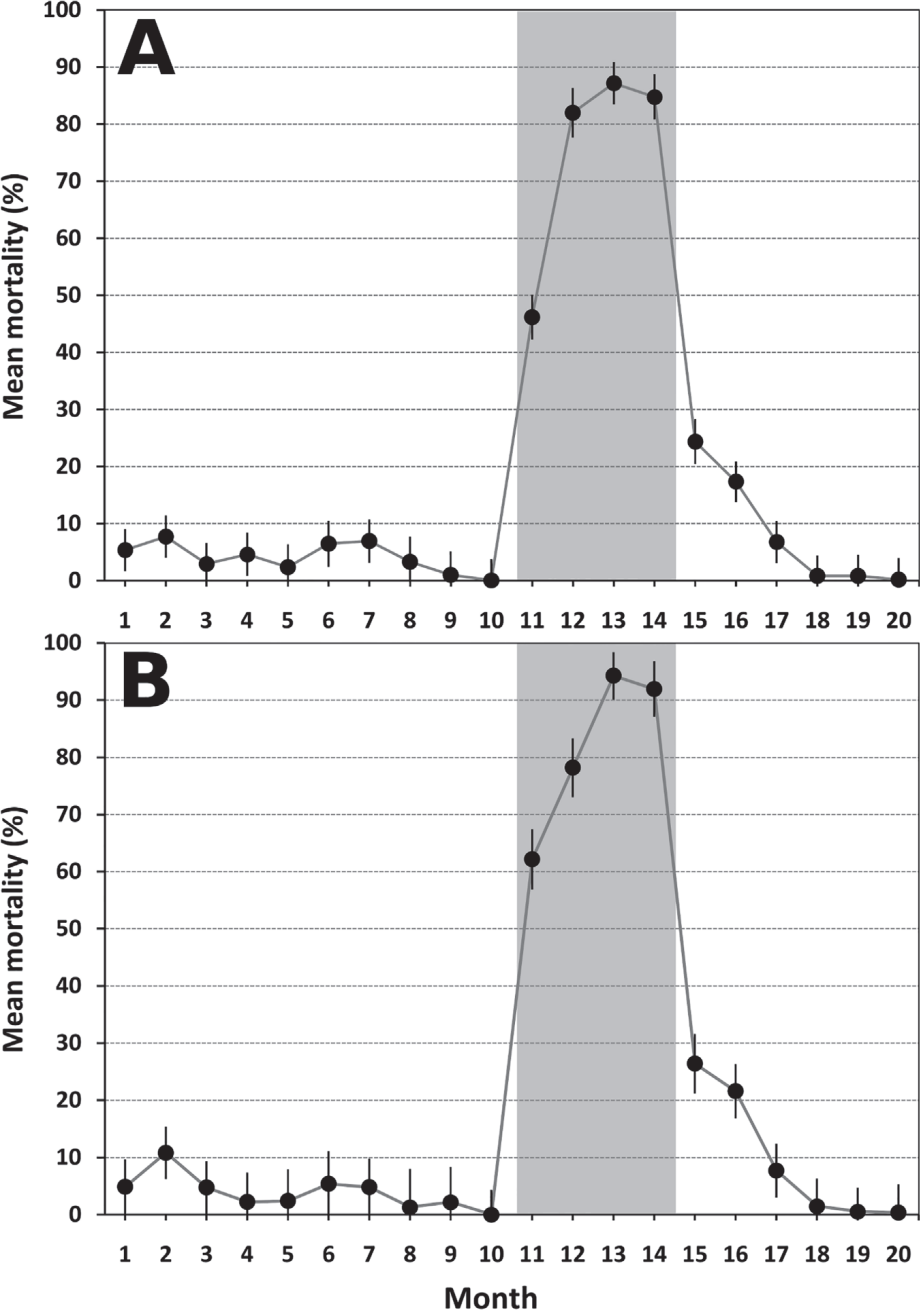
Mosquito-disseminated pyriproxyfen boosted juvenile mosquito mortality. A, species-pooled monthly mean juvenile mosquito mortality in the 2,287 sentinel breeding sites (SBSs) that contained ≥1 mosquito larva. B, *Aedes aegypti* monthly mean juvenile mortality in 1,224 SBSs with ≥1 *Ae*. *aegypti* larva. SBSs were deployed before (months 1–10), during (months 11–14; shaded), early after (months 15–17), and late after the trial (months 18–20). For each month, black circles are the mean of mortality rates across SBSs, and error bars are 95%Cis.

Fig 5A shows monthly mean juvenile mortality (all species pooled) in SBSs that contained ≥1 mosquito larva. Fig 5B shows results for *Ae*. *aegypti* (*n* = 1,224 SBS-months): juvenile mortality reached 94.3% (95%CI 90.1–98.4%) in month 13, when 430 individuals in 124 SBSs were scored for mortality or emergence. Again, these large differences among periods were highly significant (Kruskal-Wallis *P*<0.0001).

**Fig 6:**
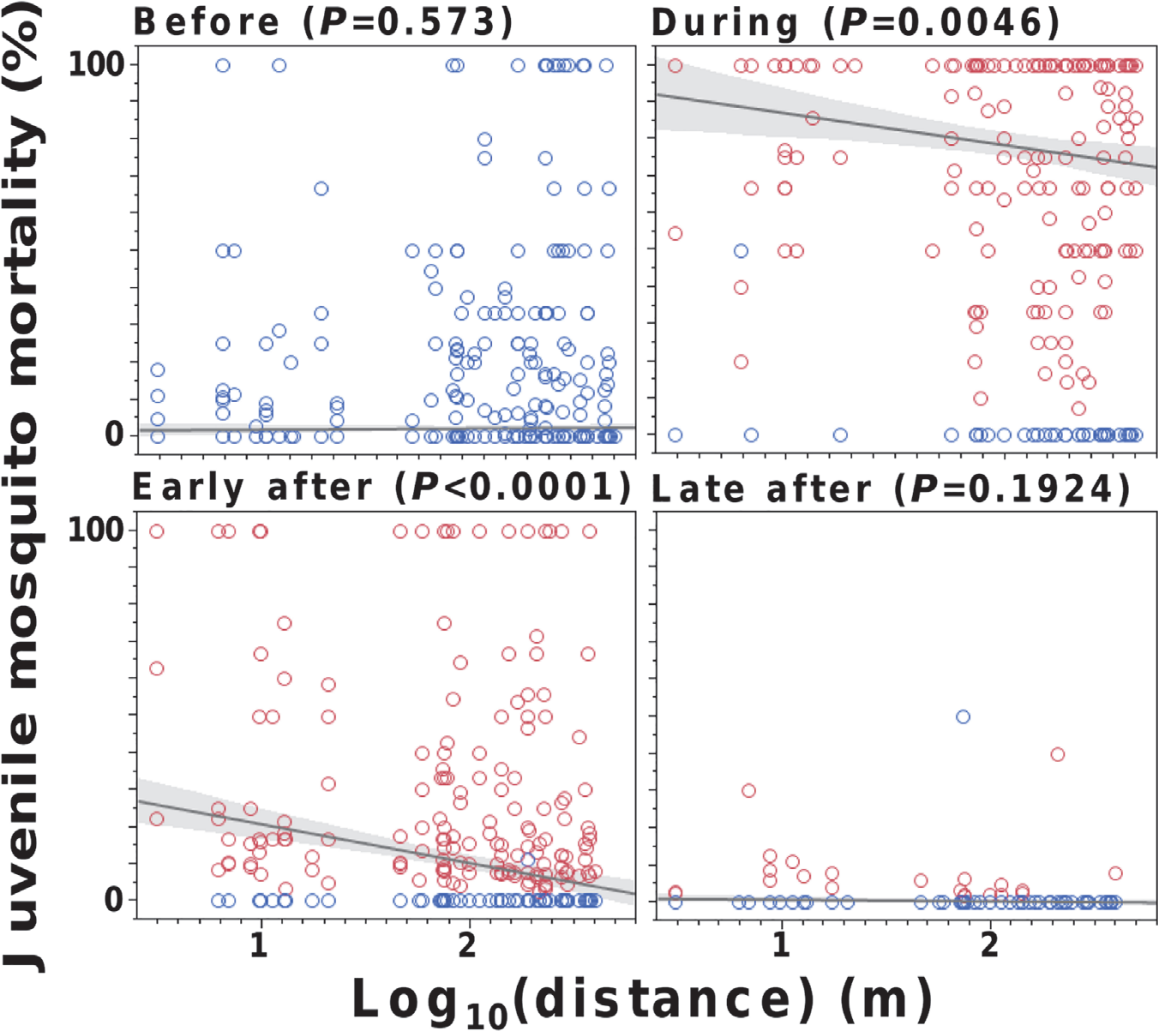
Pyriproxyfen-induced juvenile mosquito mortality decreased with distance between sentinel breeding sites (SBSs) and the nearest site of deployment of a dissemination station (DS), particularly early after DS removal. Linear regressions (with shaded 95%CIs and *P*-values) of observed mortality in SBSs *vs*. log_10_distance (m), weighted by the number of mosquitoes in each SBS. Each data point is one SBS (red, with evidence of contamination; blue, without such evidence). Note that 10 months of data were available before, four months during, and three months in both the early and late after the trial periods.

Finally, juvenile mosquito mortality decreased with increasing distance between DSs and SBSs during and, especially, early after the trial; on the contrary, no significant distance effects were evident before or late after DS deployment (Fig 6). Although larger and more persistent effects were apparent in and near the DS-deployment sub-area, the rise of mortality was evident throughout the study area, particularly for *Ae*. *aegypti* (see S2 Fig).

### Adult mosquito emergence

The median number of mosquitoes that completed development in SBSs each month before PPF dissemination was 1,177 (IQR 851–1,427), as compared to just 107 (IQR 71.8–201.5) during the trial (see S1 Dataset). Adult mosquito emergence rose back to 1,408 (IQR 972–1,910) early after the trial and, somewhat surprisingly, peaked late after DS removal to 3,435 (IQR 3,270–4,000) (Kruskal-Wallis χ^2^ = 840.2, d.f. = 3, *P*<0.0001; see S1 Dataset). For a more general comparison, the median monthly number of immature *Aedes* spp. collected in SBSs in the same area and dwellings over the 28 months preceding the present study was 2,481 (IQR 1,556–2,811) (see S1 Text); at a 4% typical baseline rate of juvenile mortality, monthly adult emergence can be estimated as about 2,400 (IQR 1,500–2,700). Overall, then, monthly adult mosquito emergence from SBSs was reduced by over one order of magnitude during the trial.

## Discussion

This paper shows that urban mosquitoes can be very effective at transferring pyriproxyfen dust-particles from simple dissemination stations to sentinel breeding sites at the neighborhood scale. All surveillance dwellings and most SBSs had evidence of contamination with mosquito-disseminated PPF at some time-point during the trial. This dramatically increased juvenile mosquito mortality in SBSs, leading to a >10-fold decrease of adult mosquito emergence. These findings confirm previous encouraging results from laboratory assays and small-scale field trials [10,11,14–16] and demonstrate that mosquito behavior can effectively be harnessed to disseminate insecticides at the neighborhood scale in real-life settings. This approach has the potential to substantially enhance mosquito control and mosquito-borne disease prevention, particularly in urban settings.

Female mosquitoes have evolved to maximize their efficiency at locating and reaching suitable breeding sites. One egg-laying strategy, displayed by *Ae*. *aegypti* and other species that breed in small containers, is to visit several distinct sites to lay a few eggs in each. This ‘skip oviposition’ behavior [12,13] may have helped increase coverage of SBS contamination with mosquito-disseminated PPF in our trial. Our results show that very simple PPF-dusted DSs can result in highly effective mosquito-driven dissemination; emulsifiable-formulation sprays seem to be less effective [17], perhaps because mosquitoes are more likely to pick PPF particles on the dusted cloth of DSs than on sprayed surfaces. Furthermore, our DSs were deployed at sheltered sites to protect them from rain or direct sunlight and were re-dusted periodically, which probably increased dissemination efficacy [17]. These findings signal potentially important operational details for PPF-based interventions. Our data also suggest that *Ae*. *aegypti* may be more efficient at disseminating PPF than *Ae*. *albopictus* or *Culex* spp. (S1 Table). Interventions based on mosquito-disseminated PPF might therefore be less effective when *Ae*. *aegypti* is absent (e.g., ref. [17]).

Low breeding-site coverage is a major shortcoming of current mosquito control strategies [9,10,22]. Thus, dengue vector control campaigns based on breeding-site detection and elimination had negligible effects on dwelling infestation by *Aedes* spp. in our study setting [9] and elsewhere in Brazil [23]. Together with the negatively-biased house infestation indices provided by routine breeding-site surveillance, this suggests that vector control agents overlook many *Aedes* breeding sites while inspecting premises [9,24]. Achieving adequate coverage is even more challenging for *Ae*. *albopictus*, which can breed in natural sites such as tree holes or epiphytic bromeliads that routine vector control does not target [25,26]. This is particularly relevant in the current context of chikungunya virus reemergence and fast spread from Africa into Asia, Oceania, Europe, and, more recently, the Americas [1,2,5,27,28; www.cdc.gov/chikungunya/]. Chikungunya transmission by *Ae*. *albopictus* can be very efficient and has been reported from several settings [28,29]. By enhancing coverage of natural breeding sites, mosquito-disseminated PPF could help control this vector species and perhaps contribute to containing the expansion of chikungunya fever. The approach, in sum, provides a powerful tool to increase breeding-site coverage, extending larvicide dissemination to sites that control agents would never detect or treat, such as sites inside closed premises or most natural breeding sites; at the same time, conspicuous breeding sites such as water tanks or catch basins could be treated directly by vector control staff following standard practice. Mosquito-disseminated PPF could therefore substantially enhance current vector control tactics, not only for dengue and chikungunya [3,6,8,10] but also for other mosquito-borne pathogens including West Nile virus (see below).

We note that our trial was conducted in an open area within a neighborhood and city where mosquitoes are widespread pests. Adult mosquitoes from adjacent, untreated areas could therefore freely migrate into the trial area and replace local recruitment that was lost due to PPF; this limited our ability to investigate the broader impact of the trial on the local mosquito population. Still, even under those circumstances, we detected a measurable decline of infestations at the dwelling level (i.e., the presence of ≥1 larva in any of a dwelling’s SBSs) by *Culex* spp. and, to a lesser extent, *Ae*. *albopictus* after DS deployment (see below and S3 Fig). The number of immature mosquitoes collected in SBSs also decreased during DS deployment and recovered afterwards (see S4 Fig). On the other hand, immigrating adult mosquitoes may have helped increase PPF coverage of SBSs and surveillance dwellings. Our results also suggest that *Ae*. *aegypti* females, thought to be poor fliers [30,31], can carry PPF over distances up to 400 m across a neighborhood where suitable breeding sites are readily available [32,33]. This dispersal ability has implications for understanding local dengue-spread dynamics [32–34] and suggests that a productive breeding focus in a non-treated premise can act as the source of adult mosquitoes for an area of about 50 ha.

Because we set clean SBSs each month, we did not assess the residual effects of PPF, which may have been important in breeding sites we did not monitor [35]. For example, observed dwelling-level infestation (i.e., presence of at least one larva in at least one SBS) by *Culex* spp. steadily fell from 30.1% at baseline to 9.3% during, 6.9% early after, and 2.5% late after the trial (see S3 Fig). We speculate that this might reflect persistent contamination of some key *Culex* breeding sites in the study area, hinting at the potential of mosquito-disseminated PPF for the control of West Nile virus- or filariae-transmitting *Culex* spp. In addition, the fact that many SBSs became contaminated after DSs were removed indicates that PPF particles persisted in the environment and were still being disseminated several months after the trial ended. Early after the trial, this might be related to mid-term PPF carriage by mosquitoes that picked dust particles at DSs but only lost them after several ovipositions. However, because of the short lifespan of non-diapausing adult mosquitoes [36], long-term PPF carriage cannot explain SBS contamination events late after DS removal. The contamination of resting sites by PPF-carrying mosquitoes, with dust particles then picked-up and secondarily disseminated by other mosquitoes, is more likely in these cases. Environmental persistence is nonetheless expected to be short, because PPF degrades quickly [37]; interestingly, over the last eight months of our study the fraction of SBSs with evidence of PPF contamination closely matched the expected fraction of PPF particles remaining active in the environment, given a 30-day half-life [37]: linear regression, R^2^ = 0.91, *P* = 0.0003 (S5 Fig).

Mortality of juvenile mosquitoes in SBSs with evidence of PPF contamination was within the range reported from small-scale [10,11,16] and direct-impact PPF trials [17]. Our results are unique because they indicate that mosquito-disseminated PPF increased juvenile mortality and reduced adult emergence at the neighborhood scale, with each of these metrics changing by over one order of magnitude. Importantly, these effects came about in spite of (i) mortality reaching 100% in just over 50% of contaminated SBSs, likely because of PPF under-dosage; (ii) substantial, yet incomplete, breeding-site coverage (as measured by SBS coverage); and (iii) the fact that the trial was conducted during the rainy-cool season, when the availability of breeding sites is at its peak and *Aedes* populations are unlikely to undergo local extinctions [9]. This approach can therefore be expected to perform well even under constraints and imperfections that are typical of real-life vector-control campaigns. We note, in addition, that we did not investigate the effects of PPF on adult mosquitoes (e.g., malformations, shorter lifespan, or reduced fertility), and that some dead early-stage larvae may have been scavenged by other larvae before SBSs were checked in the laboratory [10,14,16]. Therefore, our results likely underestimate the impact of the trial.

A key limitation of our study is that we lacked the technical means to measure the minute, parts-per-billion concentrations of PPF that kill mosquito juveniles; hence, and as in previous trials (e.g., [10,11]), we lack direct evidence of PPF contamination in our SBSs. Still, we think it extremely unlikely that our observations might stem from any unmeasured event. Evidence of PPF-induced mortality was assessed by a researcher with broad experience in the appraisal of juvenile mosquito development and morphology in PPF laboratory and field trials [10,16]. This evidence (S1 Fig) was only recorded after DS deployment. In our pre-trial laboratory tests, PPF-treated and PPF-untreated larvae had the expected morphology and could be unambiguously identified (pers. obs.; S1 Fig). Further, the dramatic increase of juvenile mortality at the time of DS deployment (Figs 5, 6 and S2 Fig) can hardly be explained by any alternative phenomenon. Not only there was an abrupt leap in mortality as the trial started: mortality also declined with distance from DSs during and early after the trial, and gradually fell back to baseline values when the DSs were removed from the environment (Figs 5, 6 and S2 Fig). We had monitored these local mosquito populations for years before this trial and never recorded any such sudden demographic shift (e.g., [9,38]). Finally, longitudinal contamination data in individual SBSs provided no evidence of either laboratory contamination (non-contaminated SBSs were recorded every month) or persistence of contamination from one month to the next (>70% of SBSs scored as contaminated in month *m* were scored as *not* contaminated at least once in month *m*+1; see S1 Text). Thus, mosquito-driven PPF dissemination is by far the best explanation available for our findings.

### Conclusions

Our results provide evidence that urban mosquitoes can be very effective at transferring PPF dust-particles from simple dissemination stations to artificial breeding sites at the neighborhood scale. Maximum monthly coverage was 94.3% for SBSs and 100% for surveillance dwellings over 50 ha, and juvenile mosquito mortality reached 87.9% in SBSs contaminated by PPF-disseminating mosquitoes. This resulted in a >10-fold rise of juvenile mosquito mortality and a >10-fold fall of adult mosquito emergence; by lowering vector/host ratios, these strong effects can help reduce the risk of arboviral disease outbreaks [39]. We conclude that this approach is a very promising complement to current mosquito control strategies, which heavily rely on the difficult task of detecting vector breeding sites and therefore perform poorly. Mosquito-disseminated insecticides could profitably be combined both with current, standard control practices and with novel, more sophisticated tactics involving transgenic or *Wolbachia*-infected mosquitoes [40–43].

## Supporting Information

S1 Dataset Raw data: mosquito presence, pyriproxyfen dissemination, and juvenile mosquito mortality at the dwelling- and breeding site-levels, plus monthly summaries of juvenile mosquito mortality and adult mosquito emergence. (XLS)

S1 Text Additional results and methodological details. (PDF)

S1 Table Mosquito-disseminated pyriproxyfen in sentinel breeding sites with and without *Aedes aegypti*, *Ae*. *albopictus*, and *Culex* spp. larvae during the trial. (PDF)

S2 Table Differences in mean *Aedes aegypti* mortality among study periods and Tukey ‘honestly significant difference’ (HSD) test. (PDF)

S3 Table Differences in mean mosquito mortality (all species pooled) among study periods and Tukey ‘honestly significant difference’ (HSD) test. (PDF)

S1 Fig The effects of pyriproxyfen (PPF) contamination on immature *Aedes aegypti* morphology. From left to right: normal fourth-stage larva; fourth-stage larva reared in water with PPF; normal pupa; and pupa reared in water with PPF (scale bars = 1 mm).
(TIFF)

S2 Fig Mosquito-disseminated pyriproxyfen boosted juvenile mosquito mortality at the neighborhood scale. Contour plots of observed juvenile mosquito mortality in sentinel breeding sites with at least one larva. Upper row, all mosquito species pooled; lower row, *Aedes aegypti*. A to D, periods before, during, early after, and late after the trial, respectively. The intervention sub-area is outlined in white in the first panel. The scale bar shows the color code of percentages; 100 contours were used. Surveillance dwellings were omitted for clarity (see Figs 1 and 2 in the main text).
(TIF)

S3 Fig Observed dwelling-level infestation by *Aedes aegypti*, *Ae*. *albopictus*, and *Culex* spp. before (B), during (D), early after (EA), and late after (LA) deployment of pyriproxyfen dissemination stations. In any given month, a dwelling was considered as infested when at least one larva was present in at least one sentinel breeding site. Error bars are 95%CIs; asterisks highlight significant differences at the 5% level.
(TIFF)

S4 Fig Number of immature mosquitoes collected in individual sentinel breeding sites (SBSs) across the 20 months of the study. Each bar corresponds to one SBS-month; note that >100 larvae were collected in one SBS (arrow) before deployment of pyriproxyfen dissemination stations (DS). The four-month trial period is highlighted.
(TIFF)

S5 Fig The fraction of sentinel breeding sites (SBSs) with evidence of contamination with pyriproxyfen (PPF) closely matched the expected fraction of PPF particles remaining active in the environment, given a 30-day half-life: scatterplot and linear regression. (TIFF)
