# Supplementary material for: Mosquito-Disseminated Pyriproxyfen Yields High Breeding-Site Coverage and Boosts Juvenile Mosquito Mortality at the Neighborhood Scale
Source: PLoS Negl Trop Dis. 2015 Apr 7;9(4):e0003702. doi: 10.1371/journal.pntd.0003702 (PMC4388722; doi:10.1371/journal.pntd.0003702)
Supplement: S1 Text — (PDF) [file pntd.0003702.s002.pdf]

Text S1. Additional results and methodological details

1. Efficacy of pyriproxyfen (PPF) used in the field trial: double-blind, randomized, controlled laboratory trial

We prepared a solution of PPF (0.05 ppm a.i. in tap water); tap water without PPF was used as the control. Treatment solution and control water were then transferred to coded glass bottles wrapped in aluminium foil. Six hundred *Aedes aegypti* larvae were lab-reared on TetraMin fish food from eggs collected at the study neighborhood. When they reached stage III, larvae were allotted to 30 independent cohorts of 20 individuals each. Each cohort was placed in an individually-coded, 200-ml white plastic cup; these cups were randomly assigned to the treatment or control group (15 cohorts each). Cups in the treatment group received 100 ml of PPF solution and cups in the control group 100 ml of water. The researcher involved in this procedure was blinded to bottle contents; treatment solution and control water did not differ in appearance or smell. A pinch of TetraMin was added to each cup, and cups were capped with gauze. Larvae were then monitored daily over three weeks by another researcher, who was blinded to the group (treatment or control) assignment of the cups. The numbers of dead juveniles (larvae or pupae) and emerging adult mosquitoes in each cohort were recorded every day. A researcher not involved in any of these procedures randomized bottles/cups and kept the codes in a closed, opaque envelope until the end of the trial. The results are summarized in the following Table.

Text S1 Table 1. Efficacy of pyriproxyfen (PPF) used in the field: results of a double-blind, randomized, controlled laboratory trial

Group	Cohorts	Initial *N*	Dead larvae	Dead pupae	Dead juveniles (%)	Adults
Treatment	15	300	0	1	1 (0.33)	299
Control	15	300	9	291	300 (100)	0
Total	30	600	9	292	301 (50.17)	299

2. Comparisons of generalized linear models based on second-order Akaike's information criterion (AICc) values

AIC combines likelihood and information theories to identify, within a given set of models, those with a better compromise between fit (as measured by the likelihood) and complexity (as measured by the number of estimable parameters). AICc also includes a finite-sample correction term; for each model M, AICc is given by the expression

$AICc = -2 \ln(L_M) + 2K + [(2K (K + 1)) / (n - K - 1)]$,

where L_M is the likelihood of the data given the model, K is the number of estimable parameters in the model, and n is sample size. For each model i, we calculated the variation in AICc relative to the best-ranking (lowest AICc) model ($\Delta AICc = AICc_i - AICc_{min}$); ΔAICc values >10 indicate that there is essentially no empirical support for the lower-ranking model (see ref. [20] of the main text for details).

Text S1 Table 2. Dwelling-level analyses: AICc-based model comparisons

Model covariates	AICc	ΔAICc	*K*	−LL
Period + Distance + Number of SBSs	470.81	0	7	217.67
Period + Number of SBSs	482.68	11.87	6	226.05
Distance + Number of SBSs	1315.07	844.26	4	644.93
Number of SBSs	1318.36	847.55	3	649.75
Null (intercept-only)	1317.89	847.08	1	655.23

AICc, second-order Akaike information criterion; ΔAICc, difference between each model's AICc and the top-ranking model's AICc; *K*, number of estimated parameters; −LL, negative log-likelihood; SBSs, operational sentinel breeding sites; see main text for further details

Text S1 Table 3. Sentinel breeding site-level analyses: AICc-based model comparisons

Model covariates	AICc	ΔAICc	*K*	−LL
Period + Distance	995.70	0	5	483.53
Period	1044.48	48.78	4	510.70
Distance	2544.13	1548.43	2	1246.57
Null (intercept-only)	2556.95	1561.25	1	1274.45

AICc, second-order Akaike information criterion; ΔAICc, difference between each model's AICc and the top-ranking model's AICc; *K*, number of estimated parameters; −LL, negative log-likelihood; see main text for further details

3. Monthly number of juvenile *Aedes* spp. collected in sentinel breeding sites (SBSs) over the 28 months preceding the current study

Below we provide a summary of the number of *Aedes* spp. larvae collected over 28 months of monitoring before the start of the present study. The area, dwellings, and sampling scheme were essentially the same as described in the main text; up to three SBSs were operated during six days per month in each surveillance dwelling.

Text S1 Table 4. Summary statistics of the monthly number of immature *Aedes* spp. collected in the same area and dwellings over the 28 months preceding the study

Species	Minimum	Quartile (25%)	Median	Quartile (75%)	Maximum
Aedes aegypti	251	1200.75	1556	2066.25	5828
Aedes albopictus	134	356.75	775.5	1026.5	2319
Overall	385	1556.25	2481	2810.75	8147

4. Persistence of contamination from one month to the next in individual sentinel breeding sites (SBSs)

Data from 167 individual SBSs were used for this assessment, which spans the six months during and the ten months after the trial. Those 167 SBSs (i) were scored as contaminated in at least one month (denoted *m*) and (ii) could also be scored as contaminated or not contaminated in the next month (*m*+1), because they did not go missing, were not overturned, did not correspond to a closed dwelling, and contained at least one mosquito larva at month *m*+1. Out of these 167 individual SBSs, 120 (71.9%) were scored at least once as contaminated at month *m* but as not contaminated at month *m*+1. Overall, we recorded 153 such events of 'month-to-month decontamination' between months *m* and *m*+1. Three events were recorded in four individual SBSs, two events in 25 individual SBSs, and one event in 91 individual SBSs.
