# Supplementary material for: Mosquito-Disseminated Pyriproxyfen Yields High Breeding-Site Coverage and Boosts Juvenile Mosquito Mortality at the Neighborhood Scale
Source: PLoS Negl Trop Dis. 2015 Apr 7;9(4):e0003702. doi: 10.1371/journal.pntd.0003702 (PMC4388722; doi:10.1371/journal.pntd.0003702)
Supplement: S1 Table — (PDF) [file pntd.0003702.s003.pdf]

Table S1. Mosquito-disseminated pyriproxyfen in sentinel breeding sites with and without *Aedes aegypti*, *Ae. albopictus*, and *Culex* spp. larvae during the intervention

Species	Contamination*			OR†	95% CI		*P*-value‡
	Yes	No	%		Lower	Upper	
Aedes aegypti							
Yes	278	27	91.15	4.29	2.47	7.54	<0.0001
No	86	36	70.49	1			
Aedes aegypti only							
Yes	196	16	92.45	3.42	1.89	6.41	<0.0001
No	168	47	78.14	1			
Aedes albopictus							
Yes	149	39	79.26	0.43	0.24	0.74	0.0016
No	215	24	89.96	1			
Aedes albopictus only							
Yes	71	28	71.72	0.30	0.17	0.54	<0.0001
No	293	35	89.33	1			
Culex spp.							
Yes	22	9	70.97	0.39	0.17	0.93	0.026
No	342	54	86.36	1			
Culex spp. only							
Yes	15	7	68.18	0.35	0.14	0.94	0.03
No	349	56	86.17	1			

*Only sentinel breeding sites that had at least one larva during the intervention period.
†Conditional maximum-likelihood odds ratio.
‡Fisher's exact test, one-tailed.
