# Supplementary material for: Mosquito-Disseminated Pyriproxyfen Yields High Breeding-Site Coverage and Boosts Juvenile Mosquito Mortality at the Neighborhood Scale
Source: PLoS Negl Trop Dis. 2015 Apr 7;9(4):e0003702. doi: 10.1371/journal.pntd.0003702 (PMC4388722; doi:10.1371/journal.pntd.0003702)
Supplement: S2 Table — (PDF) [file pntd.0003702.s004.pdf]

Table S2. Differences in mean *Aedes aegypti* mortality among trial periods and Tukey 'honestly significant difference' (HSD) test

Period (A)	Period (B)	Difference (A−B)	SE	95% CI		HSD *P*-value
During	Before	0.795	0.015	0.756	0.835	<0.0001
During	Early after	0.656	0.020	0.605	0.707	<0.0001
During	Late after	0.828	0.019	0.780	0.877	<0.0001
Early after	Before	0.139	0.017	0.095	0.184	<0.0001
Early after	Late after	0.172	0.021	0.119	0.225	<0.0001
Before	Late after	0.033	0.017	−0.009	0.076	0.1889
