# Supplementary material for: Mosquito-Disseminated Pyriproxyfen Yields High Breeding-Site Coverage and Boosts Juvenile Mosquito Mortality at the Neighborhood Scale
Source: PLoS Negl Trop Dis. 2015 Apr 7;9(4):e0003702. doi: 10.1371/journal.pntd.0003702 (PMC4388722; doi:10.1371/journal.pntd.0003702)
Supplement: S3 Table — (PDF) [file pntd.0003702.s005.pdf]

Table S3. Differences in mean mosquito mortality (all species pooled) among trial periods and Tukey 'honestly significant difference' (HSD) test

Period (A)	Period (B)	Difference (A−B)	SE	95% CI		HSD *P*-value
During	Before	0.709	0.013	0.676	0.742	<0.0001
During	Early after	0.592	0.016	0.551	0.633	<0.0001
During	Late after	0.744	0.016	0.703	0.785	<0.0001
Early after	Before	0.117	0.013	0.082	0.151	<0.0001
Early after	Late after	0.152	0.016	0.109	0.194	<0.0001
Before	Late after	0.035	0.013	0.001	0.070	0.0434
