# Supplementary figures and images for: Mosquito-Disseminated Pyriproxyfen Yields High Breeding-Site Coverage and Boosts Juvenile Mosquito Mortality at the Neighborhood Scale
Source: PLoS Negl Trop Dis. 2015 Apr 7;9(4):e0003702. doi: 10.1371/journal.pntd.0003702 (PMC4388722; doi:10.1371/journal.pntd.0003702)

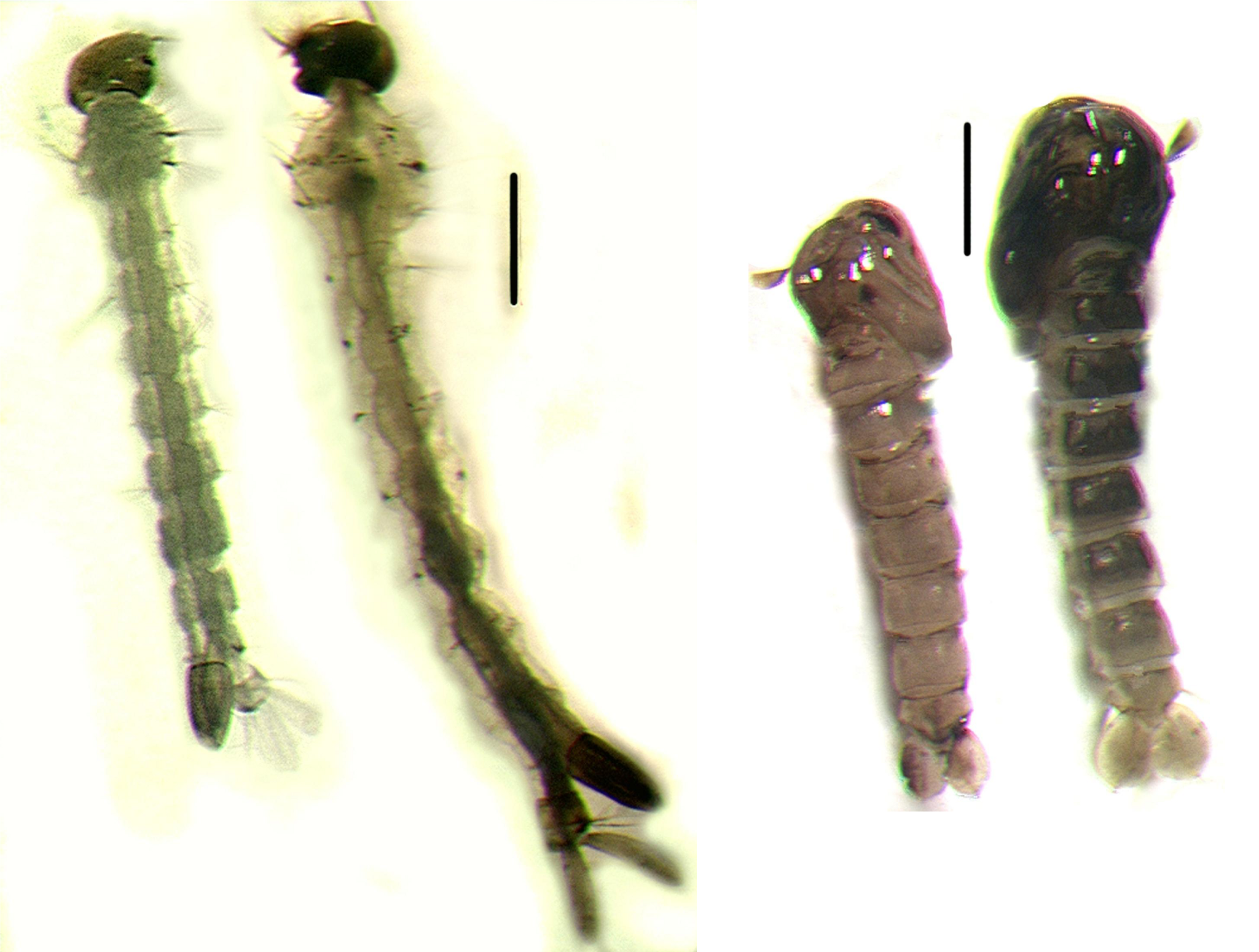

Supplement: S1 Fig — From left to right: normal fourth-stage larva; fourth-stage larva reared in water with PPF; normal pupa; and pupa reared in water with PPF (scale bars = 1 mm). (TIFF) [file pntd.0003702.s006.tiff]

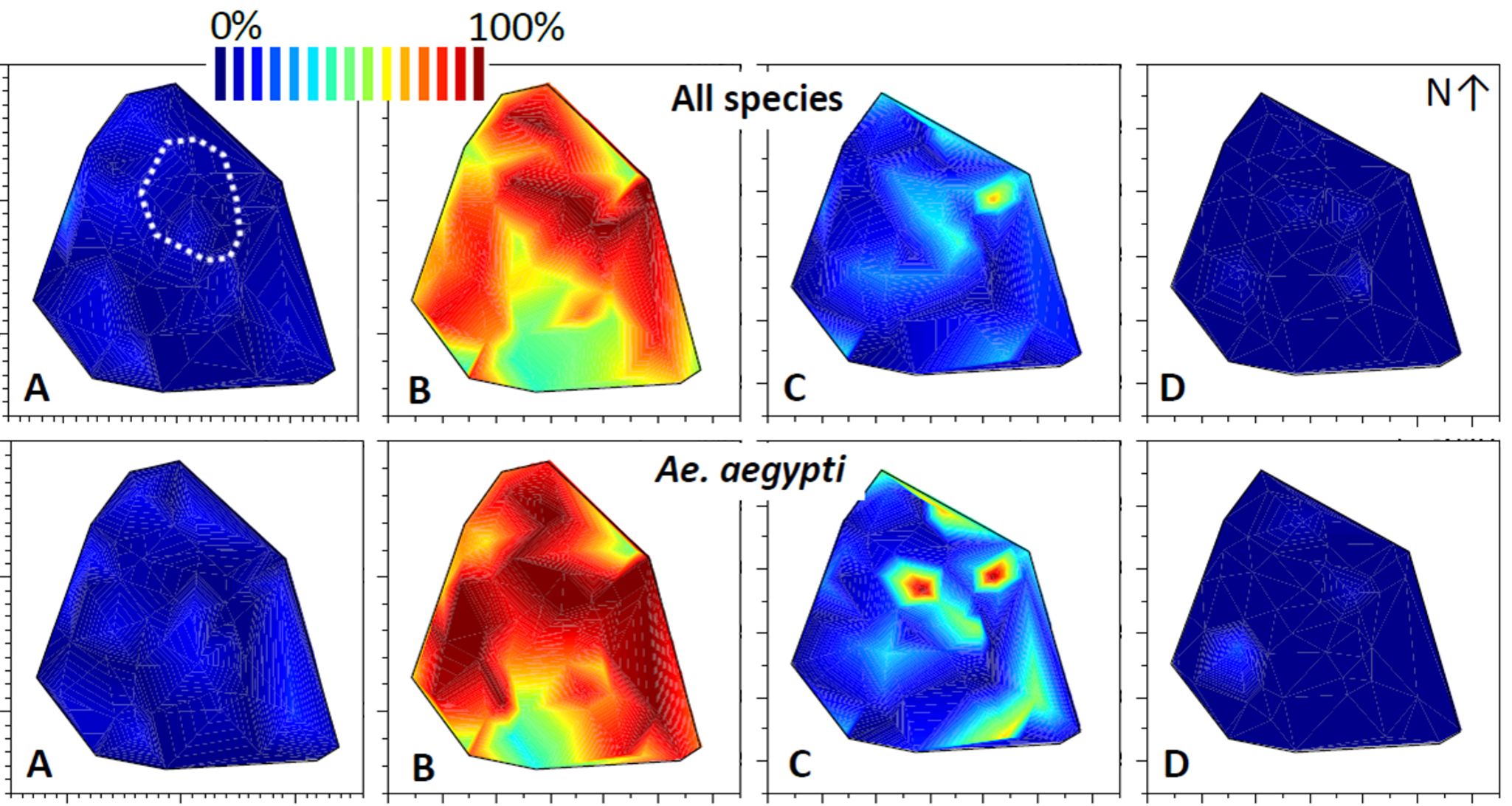

Supplement: S2 Fig — Contour plots of observed juvenile mosquito mortality in sentinel breeding sites with at least one larva. Upper row, all mosquito species pooled; lower row, Aedes aegypti. A to D, periods before, during, early after, and late after the trial, respectively. The intervention sub-area is outlined in white in the first panel. The scale bar shows the color code of percentages; 100 contours were used. Surveillance dwellings were omitted for clarity (see Figs 1 and 2 in the main text). (TIF) [file pntd.0003702.s007.tif]

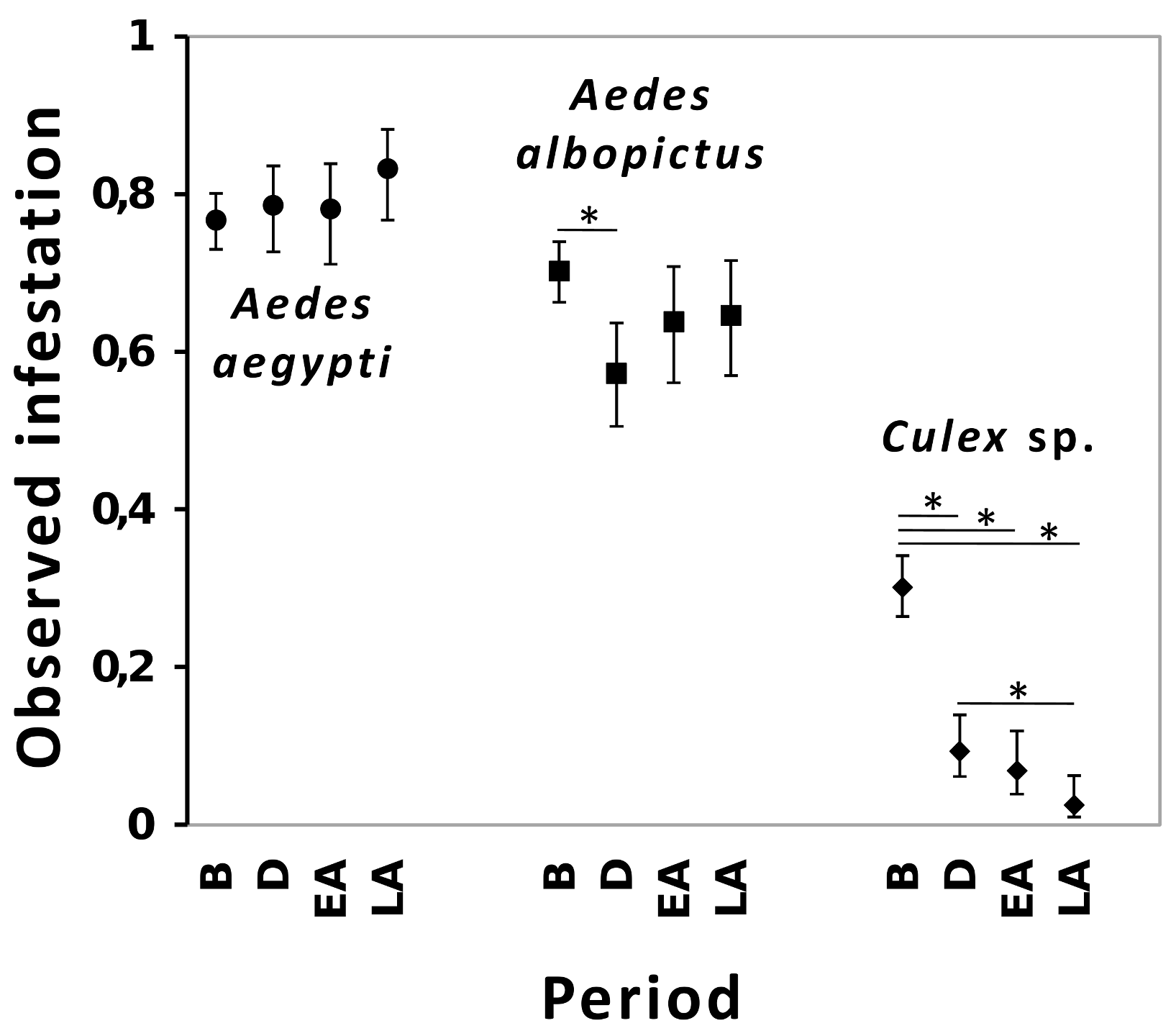

Supplement: S3 Fig — In any given month, a dwelling was considered as infested when at least one larva was present in at least one sentinel breeding site. Error bars are 95%CIs; asterisks highlight significant differences at the 5% level. (TIFF) [file pntd.0003702.s008.tiff]

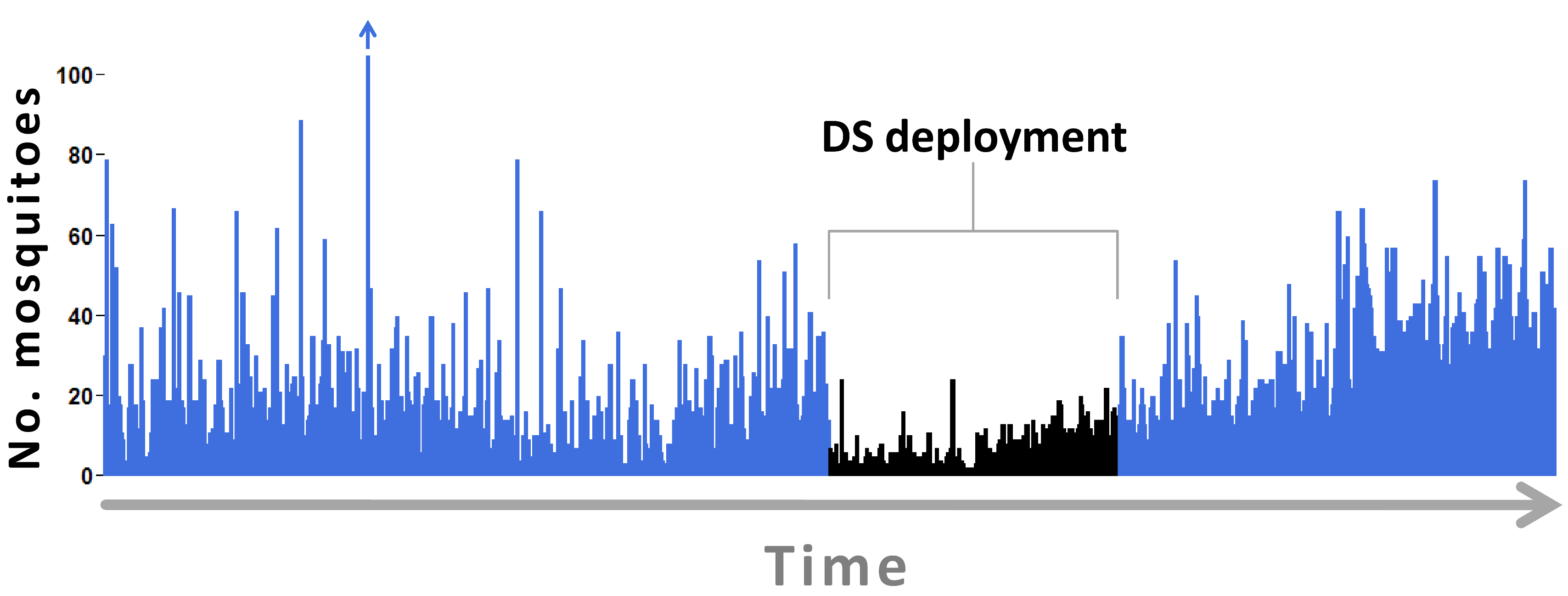

Supplement: S4 Fig — Each bar corresponds to one SBS-month; note that >100 larvae were collected in one SBS (arrow) before deployment of pyriproxyfen dissemination stations (DS). The four-month trial period is highlighted. (TIFF) [file pntd.0003702.s009.tiff]

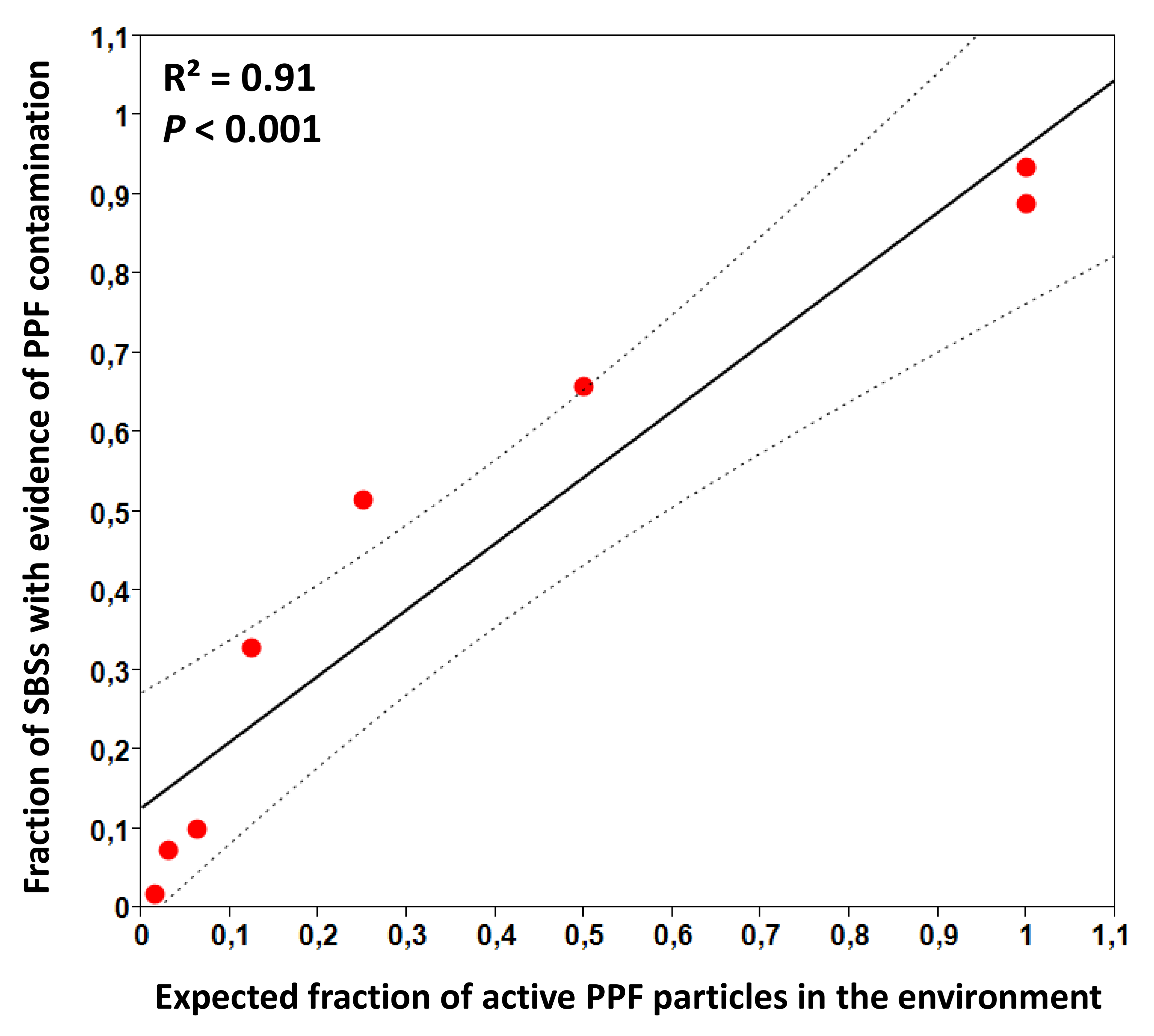

Supplement: S5 Fig — (TIFF) [file pntd.0003702.s010.tiff]
